# The Beat

**Published:** 2009-11

**Authors:** Erin E. Dooley

## Weather Comes the Flu?

Benjamin Giese et al. report in the September 2009 issue of the *Bulletin of the American Meterological Society* that the 1918 El Niño not only was much stronger than previously believed but also may have exacerbated influenza mortality that same year. The flu link seems most plausible for India, a country hit especially hard by that year’s Spanish flu pandemic, with 17 million deaths. The 1918 El Niño caused severe drought conditions in India, which contributed to a famine, weakening the population. The researchers note similar El Niño conditions are occurring this year.

## Sister Study Success

When the NIEHS-sponsored Sister Study began recruiting participants in October 2004, its goal was 50,000 participants. Five years later the study has not only met but exceeded that goal, a rarity for studies of this size. The study participants come from all 50 states and across the demographic spectrum, all having in common the fact that they had a sister with breast cancer. The 10-year prospective study will examine environmental and genetic factors that may contribute to breast cancer risk.

**Figure:**
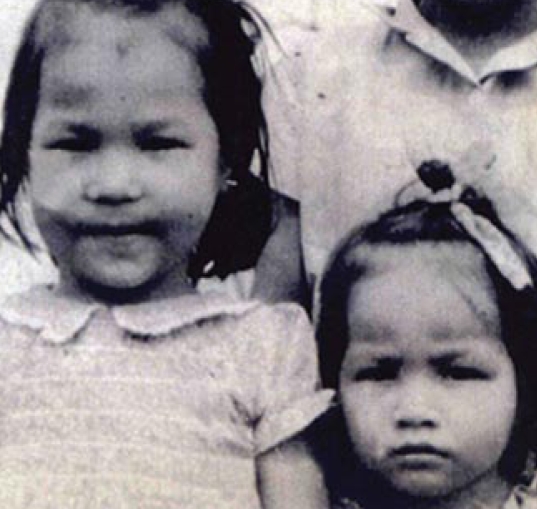
Nearly 51,000 sisters have enrolled in the Sister Study

## EPA Advises on PCBs in Caulk

In September 2009, the U.S. EPA issued a set of best practices and testing guidelines for building owners and school administrators to reduce exposure to PCBs found in caulk. PCB-laden caulk was used in buildings between 1950 and 1978, when the chemicals were phased out in the United States. The EPA also outlined research it will conduct over the next two years to help understand any health risks from PCBs in caulk; it is unclear whether or in what quantities PCBs in caulk migrate into air or dust. Findings from this research will guide the EPA in developing further recommendations to minimize exposures and safely remove the caulk. More information is available at http://www.epa.gov/pcbsincaulk/.

## Methylmercury Breakdown

In the 23 September 2009 issue of the *Journal of the American Chemical Society*, Jeremy Smith and colleagues reveal their discovery of the detailed mechanism of a specific enzyme found in bacteria, called MerB, that breaks down methylmercury. The team constructed a computational model of the enzyme’s active site and used density functional theory calculations to simulate the demethylation reaction. They found the enzyme binds to methylmercury and rearranges electrons in the compound, priming it for breakdown. The researchers hope their findings will one day be applied to ecosystem-wide remediation efforts. In 2008, 27 states issued statewide advisories for mercury in freshwater bodies.

**Figure:**
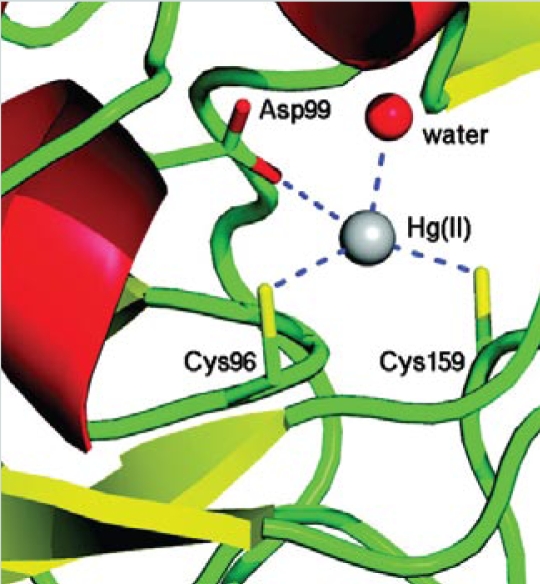
Active site of MerB

## Getting Schooled on e-Waste

In September 2009 young scholars from around the globe gathered in Eindhoven, Netherlands, for the first NVMP-StEP E-waste Summer School organized by United Nations University. The event yielded recommendations for how to put millions of unwanted electronics from developed countries to further use in classrooms and small business in developing countries—and how to plan ahead to these machines’ disposal. Participants noted it is important to reuse electronics before they are too old or damaged to be reconditioned. Some participants advocate a return deposit for e-waste to keep it from collecting unused in closets and attics.

## Cleanliness Is Next to Transparency

Two moves—one congressional, one industry-driven—are afoot to address the current lack of ingredient disclosure on household cleaning products. Trade secret protections mean only those ingredients that pose an immediate health threat are currently required to appear on product labels, but Senate and House bills currently in committee would require products to carry complete ingredient lists. Meanwhile, the industry Consumer Product Ingredient Communication Initiative is developing a uniform voluntary system for informing consumers about product ingredients—with some key exemptions—that will launch in January 2010.

**Figure:**
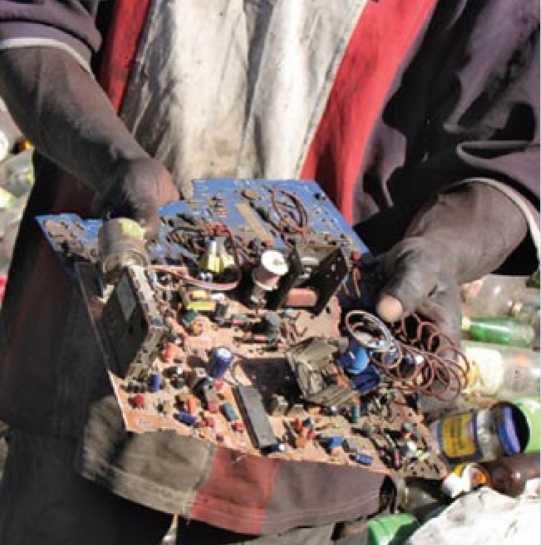
E-waste at a dump in Senegal

